# Evolution and economic evaluation of fecal incontinence management in United States intensive care units: from historical containment to automated diversion

**DOI:** 10.3389/fmed.2026.1761399

**Published:** 2026-04-20

**Authors:** Deanna Vargo, Kristin Grimes, Kiara Tickoo

**Affiliations:** ^1^Independent Researcher, Chuyahoga, Falls, OH, United State; ^2^Independent Researcher, Phoenix, AZ, United States; ^3^Consure Medical, Carrollton, TX, United States

**Keywords:** Clostridioides difficile infection, fecal incontinence, hospital-acquired pressure injuries, incontinence-associated dermatitis, stool management

## Abstract

Fecal incontinence (FI) poses a significant clinical and economic challenge in the U.S. intensive care units (ICUs), affecting 9–40% of patients and contributing to billions of dollars in healthcare costs mainly towards complications such as incontinence-associated dermatitis (IAD), hospital-acquired pressure injuries (HAPI), and Clostridioides difficile infection (CDI). This review traces the evolution of FI management from rudimentary containment methods to the newest innovative Qoramatic Automated Stool Management (ASM) system with no balloon and zero radial pressure. We compared Qoramatic ASM to traditional absorbent pads and indwelling balloon catheters (IBCs) across four patients’ subgroups Results demonstrate that Qoramatic ASM reduces per-patient care costs by 80–94.5% ($242–$1,344 vs. $1,215–$24,615 for pads/IBCs), decreases nursing time 91–96% (6–14 vs. 66–348 min/day), and nearly eliminates leakage and device-related injuries. ASM also reduces HAPI and CDI incidence, shortening hospital stays by up to 30%. Qoramatic’s improved clinical outcomes, enhanced patient dignity, and reduced staff burden positioning it is a transformative solution for FI management in ICUs, warranting broader global adoption.

## Introduction

Fecal incontinence, defined as the recurrent involuntary loss of fecal matter sufficient to impair quality of life, is a pervasive and complex challenge in the U.S. intensive care units (ICUs). Affecting an estimated 9–40% of ICU patients (1, 2) FI’s prevalence significantly exceeds that observed in acute care units (16–30%) (3) and community-dwelling adults (11–15%) (4). This high incidence underscores the unique vulnerabilities of critically ill patients, where a confluence of physiological, neurological, and environmental factors disrupts normal defecatory control.

Beyond its costly clinical implications, FI compromises patient dignity, and imposes substantial burdens on nursing staff. The management of FI in ICUs has evolved over a century, from rudimentary containment strategies to indwelling diversion systems, yet significant challenges persist in aligning interventions with the physiological realities of critically ill patients.

### Clinical significance and patient impact

FI in ICUs is not merely a clinical inconvenience but a condition with profound implications on patient’s clinical outcomes and quality of life. The involuntary loss of fecal matter erodes the patient dignity, causing psychological distress and exacerbating the emotional toll of primary critical illness. Clinically, FI is associated with a range of complications that amplify morbidity and mortality and collectively extend lengths of stay to 7 days (5), inflate national healthcare costs by an estimated $13 billion annually.^1^

Incontinence-associated dermatitis, driven by enzymatic skin maceration from prolonged fecal exposure, affects 26–46% of ICU patients with FI, increasing the risk of secondary infections and delaying wound healing (6, 7).

Hospital-acquired pressure injuries, exacerbated by shear and friction during frequent cleaning, carry a 22-fold increased risk in FI patients, contributing to prolonged hospital stays and heightened morbidity (8).

Clostridioides difficile infection, linked to fecal contamination is 40–54%, leading to extended lengths of stay and substantial treatment costs.^2^

### Pathophysiological drivers of fecal incontinence

The elevated prevalence of FI in ICUs stems from the complex pathophysiology of patient’s critical illness:

Neurological impairments, such as those resulting from stroke or traumatic brain injury, impair sphincter tone and defecatory awareness, significantly increasing FI risk.^3^ Gastrointestinal dysmotility, induced by opioid medications used for pain management and antibiotics often disrupt gut flora, alters stool consistency and increased frequency of defecation. Mechanical ventilation, a cornerstone of ICU care, elevates intra-abdominal pressures, contributing to overflow incontinence. Enteral feeding, essential for nutritional support, is further known to disrupt normal bowel function, particularly when combined with sedation, which diminishes patients’ ability to sense and control bowel movements (9, 10). Immobility, a hallmark of prolonged ICU stay, reduces peristaltic propulsion, fostering fecal stasis and increasing the likelihood of incontinence.

These factors converge to create a suitable environment for FI, particularly among patients with multiple comorbidities or those requiring invasive interventions.

### Epidemiological burden and vulnerable populations

Fecal incontinence affects an estimated 1–2 million ICU admissions annually in the United States, underscoring its significance as a public health and critical care challenge (2). The condition disproportionately impacts specific subgroups, amplifying its clinical and economic toll. These high-risk groups not only experience higher rates of FI but also suffer from its downstream consequences, including prolonged recovery times and increased healthcare costs.

Older adults (≥65 years) face substantially higher rates of fecal incontinence compared with younger adults—roughly a twofold (or greater) increase in risk—driven by age-related declines in sphincter strength and higher prevalence of comorbidities such as diabetes and neurologic disease (stroke, dementia, spinal cord injury) (11).

Diabetes is associated with an increased risk of fecal incontinence: population studies report roughly 1.5–2.0-fold higher odds of FI in people with diabetes compared with those without, and some medication effects (e.g., metformin) have been linked to nearly 2-fold higher odds of FI.^4^ Mechanically ventilated ICU patients are particularly vulnerable to acute fecal incontinence because of sedation, immobility and altered gastrointestinal motility; ICU studies report acute FI in up to ~40% of patients (2).

### Traditional FI management

#### Wipes and cotton gauze

In hospitalized, immobilized patients with fecal incontinence, nursing care traditionally relied on absorbent pads, under-garments or sheets to contain stool and protect the perineal skin until more advanced management systems were available (12). These offered minimal absorption and no protective barrier, resulting in prolonged stool-skin contact, incontinence-associated dermatitis (IAD), skin breakdown, and secondary infections. Frequent manual cleaning imposed a heavy burden on nursing staff, consuming time and diverting resources from other critical tasks in understaffed ICUs with limited infection control (13). This labor-intensive, ineffective approach amplified patient morbidity and underscored the urgent need for advanced FI management solutions.

#### Adult diapers

By the mid-20th century, adult diapers emerged as a more discreet and practical alternative, marking a modest advancement in FI management. Designed to provide a wearable containment system, adult diapers aimed to reduce the visibility of incontinence and improve patient dignity compared to the open exposure associated with gauze. However, their limited absorptive capacity, typically insufficient for high-volume or frequent FI episodes, failed to prevent leakage.

Persistent leakage perpetuated skin exposure to fecal irritants, increasing the risk of IAD and hospital acquired pressure injuries (HAPI) (14). Furthermore, adult diapers required frequent changes, contributing to a sustained high nursing workload, as staff spent considerable time managing soiled diapers plus addressing the associated skin care needs (15).

While adult diapers offered a step toward improved patient comfort and discretion, their inability to effectively manage high-volume FI highlighted the need for more robust containment strategies.

#### Open chucks and absorbent pads

From the late 1980s onward, incontinence management products evolved to include absorbent pads incorporating super-absorbent polymers, which enhanced fluid containment (including large volumes) and improved leak control compared with older fluff-pulp designs (16). They provided better skin-stool barriers, lowering IAD and complication risks. However, leakage persisted during high-volume episodes or poor positioning in non-ambulatory ICU patients, failing to eliminate skin exposure. Nursing time for changes remained high (174–348 min/day) (17), diverting resources from critical tasks like ventilation or monitoring. Despite absorptive advances, limitations in preventing skin breakdown and workload reduction highlighted the need for true stool diversion.

#### Indwelling balloon catheters (IBCs)

In the early 2000s, indwelling balloon catheters (IBCs) were introduced as an innovative attempt to achieve closed-loop stool diversion, adapting principles from Foley urinary catheters to address the shortcomings of external containment methods. IBCs were designed to be inserted into the rectum, with an inflatable balloon anchoring the device to divert stool through a narrow lumen into an external collection bag, theoretically reducing skin exposure. However, IBCs proved poorly suited to the complex anorectal dynamics of ICU patients, where variable stool consistencies (e.g., liquid diarrhea) and gastrointestinal contractions overwhelmed the catheter’s design. This resulted in significant limitations, including a 70% leakage rate, 28% expulsion rate, and 7–28% incidence of device-related injuries, such as anal erosion and sphincter dysfunction (18, 19).

**Figure 1:**
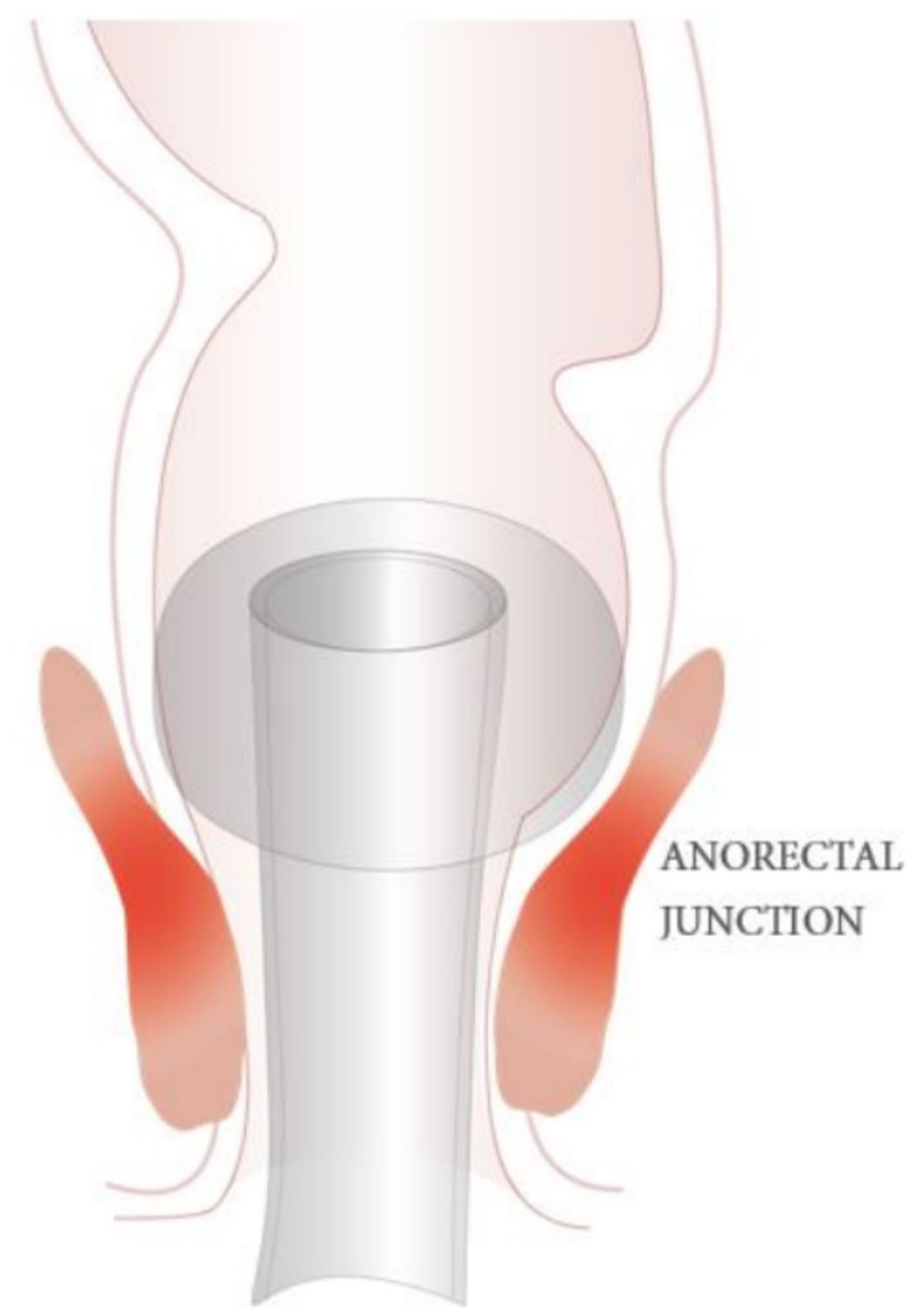
Indwelling balloon catheters anchor upon the anorectal junction.

The radial pressure exerted by the balloon caused ischemia and discomfort, further compromising patient outcomes and leading to increased morbidity. Manufacturer-led studies highlighted these issues, noting that IBCs failed to provide reliable diversion and introduced new risks, such as rectal trauma, which could exacerbate patient suffering and complicate recovery (see Figure 1).

The high failure rates and associated complications emphasized the critical need for a more effective, patient-centered solution, paving the way for newer and advanced system.

### Emergence of automated diversion: Qoramatic ASM

**Figure 2:**
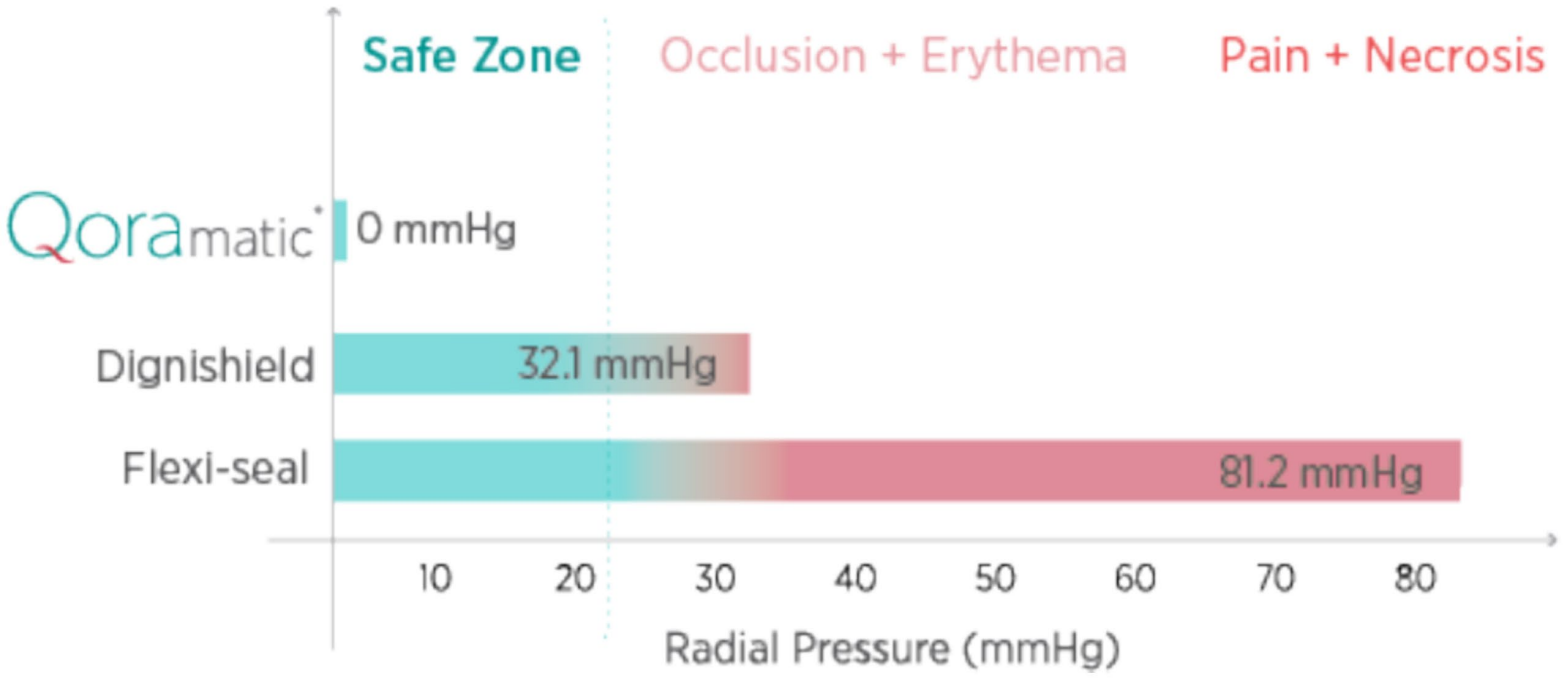
Radial pressure of IBC’s v/s Qoramatic.

The Qoramatic Automated Stool Management (ASM) system, launched across U.S. health systems in 2024, represents a transformative advancement in FI management. Drawing on technologies from advance wound care and gastrointestinal endoscopy, Qoramatic ASM employs low-grade intermittent negative pressure and automated fluid irrigation to gently divert stool into an external collection bag, without the use of a balloon. Its soft silicone receptacle employed to anchor the device within the patient rectum exerts a zero-radial pressure, which preserves sphincter integrity and motility while eliminating trauma risks (see Figure 2).

Real-world data from over 3,000 patients demonstrate remarkable outcomes: less than 1% leakage compared to 70% for IBCs (17), zero device-related injuries, and a 90% reduction in nursing time (6 min per day) (20). By minimizing enzymatic exposure and contamination, Qoramatic ASM mitigates complications and restores patient dignity, addressing a critical gap in ICU care.

### Economic and equity implications

The economic burden of FI in U.S. ICUs is staggering, with national costs estimated at $13 billion annually (see text footnote 1), driven by complications, extended lengths of stay, and intensive nursing demands.

#### Direct medical costs

Complications from FI significantly inflate healthcare expenditures:

In adult in-patients, IAD is associated with a markedly longer average hospital stay (≈ 9.7 days for incontinent patients treated for IAD vs. ≈ 6.4 days for incontinent patients without IAD treatment) and higher total index hospital costs (mean US $22,832 for incontinent with IAD treatment vs. US $16,981 for incontinent without IAD treatment) (6). Hospital-acquired pressure injuries, with a 22-fold increased risk in FI patients, cost $21,410 per case due to specialized wound care, extended hospital stays, and surgical interventions (8, 21). Clostridioides difficile infection is reported 63% in healthcare setting (see text footnote 2), costs upto $30,000 per case,^5^ reflecting diagnostic testing, isolation protocols, and antimicrobial therapy.

#### Labor costs

Nursing workload is a major driver of FI-related expenses:

Traditional management methods, such as absorbent pads, require 174–348 min per day per patient for cleaning and changes (16) costing $175–$262 per day at an average nursing wage of $45 per hour (22).

Indwelling balloon catheters (IBCs), while less labor-intensive than pads alone, still demand 66–120 min per day (16) ($50–$90) for maintenance and reinsertion due to frequent expulsion (28%) and leakage (70%) (17, 18). These labor costs, multiplied across 1–2 million FI cases, translate to millions of nursing hours annually, equivalent to approximately 43,000 nurse full-time equivalents. This diversion of nursing resources exacerbates ICU burnout (upto 50% prevalence) (23) and contributes to workforce shortages, further inflating costs through overtime and agency staffing.

FI-related complications extend ICU lengths of stay by 4 to 19 days, significantly increasing costs. For uncomplicated FI, the average length of stay is 4 days,^6^ but this rises to 11 days for patients with CDI (24), and 19 days for those with HAPI (25).

Using 2025 U.S. hospital cost estimates indexed to the Consumer Price Index and Centers for Medicare & Medicaid Services Diagnosis-Related Group weights, each additional ICU Day costs approximately $2,500–$4,000, excluding complication-specific treatments.

For high-risk subgroups, additional surcharges for personal protective equipment and wound care add $500–$1,000 per day.

### Objectives of the review

This review aims to provide a comprehensive analysis of FI management’s evolution in the U.S. ICUs, from historical containment strategies to the advancement of automated fecal diversion with Qoramatic ASM. Synthesizing the clinical, economic, and equity perspectives, it is clear that Qoramatic ASM has a transformative potential.

## Methods

The review included four health states:

*Uncontained FI*: patients with active incontinence, at risk of complications such as incontinence-associated dermatitis (IAD), hospital-acquired pressure injuries (HAPI), Clostridioides difficile infection (CDI), or other healthcare-associated infections (HAIs) due to unmanaged FI.

*Contained FI*: patients with successful stool diversion, reducing complication risks.

*Resolved FI*: patients recovering from FI without further complications.

*Death*: mortality associated with severe complications such as Clostridioides difficile infection (CDI) and hospital-acquired pressure injuries (HAPI) has been reported to range between 2 and 10%, with some studies noting higher rates depending on patient comorbidities and severity of illness (26).

### Cohort and subgroup characteristics

The synthetic cohort was designed to represent the 1–2 million U.S. ICU patients with FI annually, based on data from the Healthcare Cost and Utilization Project National Inpatient Sample (HCUP NIS) 2023. To account for clinical heterogeneity, the cohort was stratified into four subgroups based on the severity and associated complications of uncontained FI

1. Uncomplicated FI: patients experiencing 5–7 episodes per day, with an average length of stay of 4 days (23).
2. FI with CDI: patients with 12–15 episodes per day, a length of stay of 11 days (14), and additional costs for isolation protocols (see text footnote 5).
3. FI with HAPI: Patients with 5–7 episodes per day, a length of stay of 19 days (reduced to 12 days with Qoramatic ASM due to accelerated healing from reduced skin exposure) (24).
4. FI with Other HAIs: patients with 5–7 episodes per day, a length of stay of 10 days, and additional treatment costs for infections such as catheter-associated urinary tract infections or surgical site infections.

These subgroups were informed by epidemiological data and clinical observations, capturing the diverse risk profiles and cost implications of FI in ICU settings.

### Interventions compared

Three FI management strategies were compared for each subgroup:

Absorbent pads: disposable underlays with polymer gels, capable of absorbing 2–3 L of stool but requiring frequent changes and exposing skin to irritants, contributing to IAD and HAPI. [Manufacturers: Medline Industries, Kimberly-Clar, TENA, Birchlabs, and Boenmed].

Indwelling balloon catheters (IBCs): devices adapted from urinary catheters, using rectal balloon inflation at 30–80 mmHg (27) radial pressure for internal stool diversion. [Manufacturers: Convatec, Becton Dikenson, Hollister—last two companies have either discontinued or reduced the sale of IBCs due to low adoption].

Qoramatic ASM: an automated system employing low-grade intermittent negative pressure and automated saline irrigation to divert stool. [Manufacturer—Consure Medical].

### Data sources

Model parameters were synthesized from multiple high-quality sources to ensure accuracy and generalizability:

Systematic Reviews: A PRISMA-compliant review of 28 studies provided data on FI prevalence (9–40%), complication rates (e.g., 26–46% for IAD), and intervention outcomes.

Administrative Databases: HCUP NIS 2023 data informed cohort characteristics, lengths of stay, and baseline cost estimates for U.S. ICU patients.

Qoramatic: De-identified data from a prospective multicenter use of over 3,000 patients provided real-world outcomes for Qoramatic ASM, including leakage rates (<1%), injury rates (0%), and nursing time (6 min per day).

Manufacturer and Peer-Reviewed Literature: IBC performance metrics were drawn from studies by manufacturers (e.g., ConvaTec, Bard, Hollister) and independent analyses, ensuring balanced comparator data.

Costs were reported in 2025 U.S. dollars, adjusted for inflation using the Consumer Price Index and aligned with Centers for Medicare & Medicaid Services Diagnosis-Related Group weights.

### Cost estimation

Cost inputs were categorized into four components, grounded in peer-reviewed pad cost literature and meta-analyses:

*Device costs*:

1. Pads/pouches: $0.50/pad; Average use of 6–10 pads per day^7^
2. IBCs: $90–$120 per device, plus $6 per day for bags^8^
3. ASM: $175 per device, plus $10 per day for bags (19)

*Labor costs*: calculated at an average nursing wage of $45 per hour.^9^

1. Pads: 174–348 min/day for pad changes, cleaning, and repositioning (16).
2. IBCs: 66–120 min/day for insertion, monitoring, irrigation, milking and troubleshooting (16, 28).
3. ASM: 6–12 min/day for insertion, monitoring and significant time savings due to automation of irrigation and device maintenance (19).

*Modeling parameters*: several key assumptions were incorporated into the base-case analysis:

Device compliance: all devices were assumed to be applied correctly and maintained according to manufacturer instructions. Leakage & additional resources in cases of expulsion, rates were drawn from published literature (IBCs: 70%; ASM: <1%).

Length of stay: median ICU LOS of 4 days; extended up to 30 days in complication scenarios.

Complication attribution: FI was assumed to contribute directly to IAD cases, HAPI cases, and CDI cases in ICU patients, based on pooled observational estimates.

Bleeding/expulsion cost: these included costs arising from leakage-related additional pad changes, catheter expulsions, bleeding events requiring transfusions or surgery—costed per event using data from the American Association of Blood Banks (AABB)—as well as PPE and wound care consumables for infection control and HAPI management. $2,000 for IBC-related bleeding (transfusions/surgery) (29).

### Additional clinical complication cost

#### CDI subgroup (LOS: 10 days)

Pads/IBC: $500/day rationale: daily costs for PPE and infection control for CDI isolation/contact precautions, amplified by FI leakage requiring frequent changes.

The total CDI-attributable cost per case ranges from approximately $7,000–$29,000, reflecting variations in LOS (3–11 days) (24) and complexity of care. This corresponds to an average daily cost between $650 and $2,600, with the $500 figure representing a mid-range estimate for PPE and infection control expenditures in scenarios complicated by leaky FI management. Such elevated costs contribute substantially to the overall economic burden of CDI in critically ill patients and underscore the need for effective containment and prevention strategies.

#### HAPI subgroup (LOS: 19 days)

Pads/IBC: $1,000/day rationale: daily wound care supplies (dressings, barriers creams) plus wound care nurse (WOC) involvement (consultations) for stage 3/4 HAPIs worsened by FI moisture/enzymes.

Total HAPI cost $2,159–$21,410/case (over 4–19 days: ~$500–$5,000/day); $1,000 fits severe ICU stage 3/4 daily care (14).^10^

#### CAUTI/SSI subgroup (LOS: 10 days)

Pads/IBC: $550/day Rationale: Daily costs for infection monitoring/treatment (antibiotics, cultures, LOS, surgical repairs) linked to FI contamination of catheters or surgical sites.

Average CAUTI cost $10,197/case ($550 for subset like PPE/supplies) (30).

### Outcomes measured

The analysis evaluated primary and secondary outcomes to assess intervention performance comprehensively:

Primary outcomes included:

1. Total cost per FI patient per ICU stay.
2. Nursing time required per FI patient per day.
3. Incidence and additional cost with major complications (IAD, HAPI, CDI, bleeding).
4. Incremental cost-savings associated with ASM compared to traditional methods.

Secondary outcomes included infection transmission prevention, staff injury reduction, and broader system-wide cost avoidance.

### Limitations

The analysis faced several limitations:

Modeling assumptions: economic modeling relied on assumptions about complication attribution, nursing time, and costs, which introduce uncertainty. Sensitivity analyses were performed, but real-world variation may differ.

Exclusion of societal costs: analysis focused on the hospital perspective; broader costs, such as caregiver burden post-discharge or litigation, were not captured.

Geographic scope: findings are U.S.-centric; while trends are likely generalizable, absolute savings may vary in different healthcare systems.

## Results

### Integrated clinical and economic outcomes across subgroups

The CEA assessed four subgroups, characterized in Table 1: uncomplicated FI (5–7 episodes/day, 4-day length of stay), FI with Clostridioides difficile infection (CDI; 12–15 episodes/day, 11-day length of stay), FI with HAPI (5–7 episodes/day, 19-day length of stay with pads/IBCs, 12 days with Qoramatic ASM), and FI with other healthcare-associated infections (HAIs; 5–7 episodes/day, 10-day length of stay). Qoramatic ASM consistently delivered superior clinical outcomes and cost savings, integrating patient-centered benefits with economic efficiency.

**Table 1:** Additional length of stay and cost associated with hospital acquired complications.

Complication	Additional LOS (days)	Additional cost ($)
HAPI	4.31–19	$ 2,159–$ 21,410
CDI	2.95–11	$ 7,286–$ 29,000
CLABSI	8.8–10	$ 10,750–$ 23,242
CAUTI	0.4–2	$ 1,006–$10,197
SSI	4.9–10	$ 21,040–$ 34,434

#### Uncomplicated FI

Total per-patient costs were highest for pads ($1,791.84), followed by IBC ($1,568.88), and lowest for Qoramatic ($239.20). Despite the initial device cost, Qoramatic demonstrated an 86–90% cost reduction relative to traditional methods, largely due to minimal labor and leakage costs.

#### FI with CDI

For patients with concurrent CDI, total costs rose substantially across all methods, driven by longer length of stay (LOS, 11 days) and additional infection-related expenses. Pads incurred the highest total cost ($16,587.12), followed by IBC ($9,639.43), whereas Qoramatic remained significantly lower ($1,451.55). The Qoramatic method reduced overall cost by approximately 91% compared to pads.

#### FI with HAPI

This subgroup demonstrated the most pronounced cost and outcome differences among all patient categories. With an average length of stay (LOS) of 19 days for standard care and 12 days for Qoramatic use, total costs were estimated at $27,511.24 for pads and $26,077.20 for IBC, compared with only $6,367.60 for Qoramatic—a 76–77% cost reduction relative to conventional methods. The reduced LOS associated with Qoramatic can be attributed to its closed, no leakage feature, which minimizes skin exposure to effluent, thereby promoting healing of existing pressure injuries and reducing the risk of new development of injuries.

#### FI with Other HAIs

**Figure 3:**
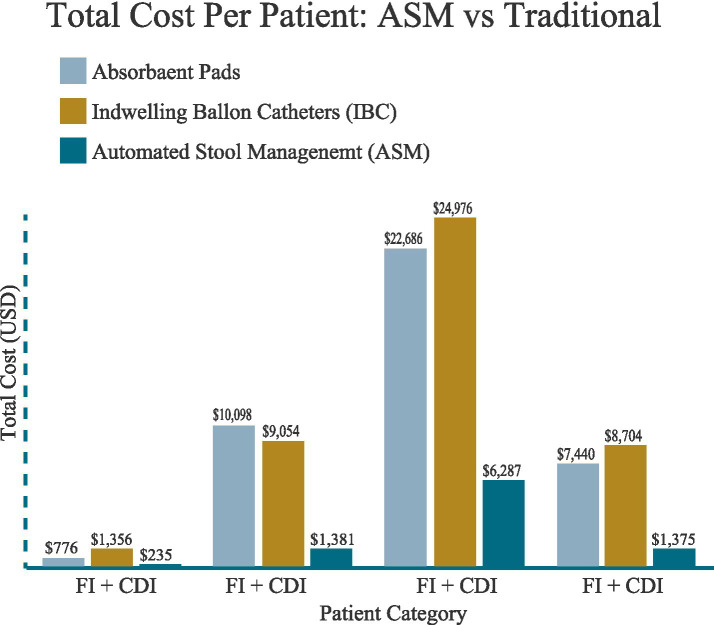
Cost per patient with FI management options.

For FI patients with other HAIs (LOS = 10 days), the total cost per patient was $9,979.60 for pads, $9,272.21 for IBC, and $1,435.50 for Qoramatic. Similar to other groups, Qoramatic achieved an 85–90% cost savings (see Figure 3; Table 2).

**Table 2:** Summary of fecal incontinence (FI) management costs by patient type and method.

Patient type	LOS (days)	Method	Average total cost ($)
Uncomplicated FI	4	**Pads**—Pad $3 + Labor, supplies $191 → **$194/day**	**776**
		**IBC**—Device $124 (fixed) + Labor $66 + Leakage/Expulsion $242 → **$308/day**	**1,356**
		**Qoramatic**—Device $215 (fixed) + Labor $5 → **$100/day**	**235**
FI + CDI	11	**Pads**—Pad $7 + Labor, Supplies $411 + Complications $500 → **$918/day**	**10,098**
		**IBC**—Device $166 + Labor $66 + Leakage $242 + Complications $500 → **$808/day**	**9,054**
		**Qoramatic**—Device $215 + Labor $5 + Leakage $1 + Complications $100 → **$106/day**	**1,381**
FI + HAPI	19 (12 for Qoramatic)	**Pads**—Pad $3 + Labor, supplies $191 + HAPI care $1,000 → **$1,194/day**	**22,686**
		**IBC**—Device $124 + Labor $66 + Leakage $242 + HAPI care $1,000 → **$1,308/day**	**24,976**
		**Qoramatic**—Device $215 + Labor $5 + Leakage $1 + HAPI care $500 → **$506/day**	**6,287**
FI + Other HAIs	10	**Pads**—Pad $3 + Labor, supplies $191 + Infection cost $550 → **$744/day**	**7,440**
		**IBC**—Device $124 + Labor $66 + Leakage/Expulsion $242 + Infection cost $550 → **$858/day**	**8,704**
		**Qoramatic** – Device $215 + Labor $5 + Leakage $1 + Infection cost $110 → **$116/day**	**1,375**

## Discussion

The management of fecal incontinence (FI) in intensive care has evolved from basic containment to closed-loop diversion, reflecting growing awareness of its clinical, economic, and operational burden. Traditional methods such as absorbent pads and indwelling balloon catheters (IBCs) remain commonly used, particularly in lower-acuity or resource-limited settings, but both have intrinsic limitations that compromise patient outcomes and efficiency. Absorbent pads, though inexpensive and easy to apply, function primarily as containment devices rather than diversion systems.

They offer minimal protection against leakage, prolong skin exposure to fecal enzymes, and significantly increase the risk of incontinence-associated dermatitis (IAD) and hospital-acquired pressure injuries (HAPI). Nursing staff spend an estimated 174–348 min daily on pad changes and perineal cleaning, contributing to labor costs of $175–$262 per day and cumulative per-patient costs exceeding $9,000–$27,000 for typical ICU stays.

The resulting workload not only affects care quality but also amplifies burnout and resource strain in critical care units.

Indwelling balloon catheters were introduced as a more “closed” solution to minimize contamination, yet their retention balloon exerts radial pressure (30–80 mmHg) on the rectal wall, predisposing to mucosal injury, bleeding, and ischemia. Leakage (70%) and expulsion (28%) rates remain high, with device-related injury reported in 7–28% of patients. While IBCs reduce daily nursing time to 66–120 min, the need for frequent reinsertion, irrigation, and monitoring offsets operational gains. Economically, total per-patient costs range from $9,272 to $26,077, only marginally lower than pads.

The Qoramatic Automated Stool Management (ASM) system marks a pivotal transition from passive containment to active diversion. Using a balloon-free, low-pressure design with intermittent suction and irrigation, Qoramatic achieves near-total stool diversion with <1% leakage and zero device-related injuries. Its average daily management time is 6–12 min, reducing nursing burden by >90% and minimizing exposure-related complications. Across subgroups, total per-patient costs range from $239.2 (uncomplicated FI) to $6,367.6 (HAPI), representing 85–91% cost reductions compared with pads or IBCs.

Although ASM demonstrates clear superiority in efficiency and safety, pads and IBCs maintain situational relevance where automated systems are unavailable or in low-resource units. The clinical context, stool volume, and patient tolerance should guide method selection. When evaluated comprehensively, automated diversion optimizes both cost and care quality, aligning with modern ICU standards for infection control and nursing efficiency.

## Conclusion

Fecal incontinence (FI) in critical care settings remains a complex and costly challenge, often associated with increased morbidity, hospital-acquired infections (HAIs), and extended length of stay. Conventional management approaches—including absorbent pads, manual cleansing, and balloon-based fecal management systems—have historically demonstrated limited effectiveness in achieving consistent containment while introducing their own clinical and operational drawbacks. These methods are frequently resource-intensive, elevate the risk of perianal injury and pressure injuries, and impose a significant burden on nursing workflow. In this context, the emergence of the Qoramatic Automated Stool Management (ASM) system represents a paradigm shift in the management of fecal incontinence among ICU patients.

The Qoramatic ASM system integrates to provide a closed, continuous, and gentle mechanism for fecal diversion and collection. By removing the need for balloon retention and reducing manual interventions, Qoramatic minimizes risks associated with tissue ischemia, leakage, and infection. Moreover, the automated design ensures optimal containment and patient comfort, reducing skin exposure to moisture and irritants—critical factors in preventing incontinence-associated dermatitis (IAD) and hospital-acquired pressure injuries (HAPI). Clinically, this translates into better patient outcomes and reduced complication rates, supporting adherence to infection prevention and patient safety protocols.

From an economic perspective, evidence from cost-minimization and cost-effectiveness analyses indicates that automated stool management offers substantial savings compared to traditional containment methods.

Operational efficiency further reinforces its clinical value. The automation of fecal management reduces nursing workload by up to 90%, freeing clinical staff to focus on higher-acuity tasks. The device’s closed system also enhances dignity, infection control, and environmental hygiene—areas often neglected in conventional methods. These improvements not only optimize bedside efficiency but also align with broader healthcare quality initiatives aimed at enhancing safety, reducing preventable harm, and promoting equitable access to advanced care technologies.

Qoramatic ASM transcends the limitations of historical FI management strategies, offering a clinically attuned and economically sustainable solution tailored to the needs of U.S. intensive care units. Its dominance in both cost-effectiveness and clinical safety positions it as the emerging standard of care in stool management. By addressing the intertwined clinical, economic, and equity challenges inherent in fecal incontinence care, Qoramatic represents more than an incremental innovation—it signifies a transformative step toward holistic critical care improvement. The compelling body of clinical and economic evidence underscores the urgency for its widespread and equitable adoption. Swift integration of automated stool management technology like Qoramatic can safeguard vulnerable ICU populations, streamline nursing operations, and strengthen the overall quality and efficiency of healthcare delivery in modern critical care settings.

## Footnotes

1
https://effectivehealthcare.ahrq.gov/sites/default/files/pdf/topic-nomination-fecal-incontinence-supporting.pdf

2
https://www.sciencedirect.com/science/article/pii/S1198743X18301459

3
https://www.researchgate.net/publication/286027407_Bowel_dysfunction_in_patients_with_brain_damage_resulting_from_stroke_and_traumatic_brain_injury_A_retrospective_study_of_a_case_series

4
https://link.springer.com/article/10.1007/s00192-015-2730-9?utm

5 https://nursingeducation.org/careers/registered-nurse/salary/?

6
https://www.mayoclinic.org/diseases-conditions/c-difficile/symptoms-causes/syc-20351691?utm_source

7
https://www.graylinemedical.com/products/bard-weber-rubber-rectal-catheter-double-lumen-design-with-balloon-30fr-single-use-non-sterile-latex

8
https://nursejournal.org/registered-nursing/rn-salary/?utm_source

9
https://www.acpgbi.org.uk/_userfiles/import/2017/02/Guidelines-for-the-use-of-faecal-management-systems-in-adult-patients-NHS-2015.pdf

10
https://institutionalrepository.aah.org/nurs/269/?utm_source
